# Evaluation of InSAR Tropospheric Delay Correction Methods in the Plateau Monsoon Climate Region Considering Spatial–Temporal Variability

**DOI:** 10.3390/s23239574

**Published:** 2023-12-02

**Authors:** Qihang Yang, Xiaoqing Zuo, Shipeng Guo, Yanxi Zhao

**Affiliations:** School of Land and Resources Engineering, Kunming University of Science and Technology, Kunming 650093, China

**Keywords:** InSAR, tropospheric delay, linear models, Generic Atmospheric Correction Online Service for InSAR, ERA-5 atmospheric reanalysis dataset

## Abstract

The tropospheric delay caused by the temporal and spatial variation of meteorological parameters is the main error source in interferometric synthetic aperture radar (InSAR) applications for geodesy. To minimize the impact of tropospheric delay errors, it is necessary to select the appropriate tropospheric delay correction method for different regions. In this study, the interferogram results of the InSAR, corrected for tropospheric delay using the Linear, Generic Atmospheric Correction Online Service for InSAR (GACOS) and ERA-5 atmospheric reanalysis dataset (ERA5) methods, are presented for the study area of the junction of the Hengduan Mountains and the Yunnan–Kweichow Plateau, which is significantly influenced by the plateau monsoon climate. Four representative regions, Eryuan, Binchuan, Dali, and Yangbi, are selected for the study and analysis. The phase standard deviation (STD), phase–height correlation, and global navigation satellite system (GNSS) data were used to evaluate the effect of tropospheric delay correction by integrating topographic, seasonal, and meteorological factors. The results show that all three methods can attenuate the tropospheric delay, but the correction effect varies with spatial and temporal characteristics.

## 1. Introduction

Interferometric synthetic aperture radar (InSAR) has been widely used in seismic inversion, urban subsidence detection, volcanic activity monitoring, etc., with centimeter- or even millimeter-level positioning accuracy and spatial resolution of up to the meter level. However, as with other astronomical and space geodetic techniques, InSAR observations are affected by tropospheric delays. For the micro-meteorological layer in the lower troposphere (<5 km), complex changes in atmospheric elements can produce atmospheric delay signals in interferograms, resulting in an error of about 10 cm in the InSAR deformation monitoring results, which poses a challenge for InSAR high-precision deformation monitoring applications [1]. In the application of seismic deformation monitoring and seismic inversion, the atmospheric delay signal often conceals the surface displacement caused by small-scale tectonic movements in a large area, which seriously restricts the accuracy of coseismic deformation observation and seismic prediction using InSAR technology [2]. In urban subsidence monitoring, the InSAR phase observation error caused by an atmospheric delay signal will ignore a slow ground-elevation deformation, which will lead to the destruction of urban roads, bridges, and dams and even lead to geological disasters. Globally, the plateau monsoon climate zone has a high potential risk of ground subsidence due to complex meteorological conditions, frequent tectonic activity, and anthropogenic activities brought about by urbanization. In addition, according to the theory of atmospheric stratification, the atmospheric delay effect can be divided into tropospheric delay and ionospheric delay. Since this study uses C-band SAR data and the study area is located in low latitudes, the ionospheric delay signal is small and negligible.

The tropospheric delay error in InSAR applications is caused by the temporal and spatial variation of atmospheric parameters in the troposphere during two acquisitions [3,4]; change of atmospheric water vapor content is the most important factor causing InSAR tropospheric delay. According to the spatial distribution characteristics of tropospheric delay, tropospheric delay can be divided into vertically stratified delay and turbulently mixed delay [5]. The vertically stratified delay is caused by the difference in the atmospheric refractive index in the vertical profile, which is strongly correlated with the terrain and only affects areas with large terrain fluctuations. Turbulently mixed delay is the result of turbulent processes in the atmosphere and is highly stochastic, affecting both flat and undulating terrain. Currently, there are four main methods for attenuating the tropospheric delay of the InSAR: (1) Temporal and spatial filtering methods that depend on time and space [6,7]. However, the use of spatial–temporal filtering is ineffective in capturing seasonal variations in tropospheric delay because seasonal variations in the water vapor content of the air introduce a systematic component to time-dependent tropospheric delay. (2) Estimation of the tropospheric delay based on global navigation satellite system (GNSS) observations [8,9]. However, this method relies on a dense GNSS network, which is often not met in the study area in general, so the method has low applicability in tropospheric delay correction. (3) Empirical model correction methods based on the analysis of the observed delayed phase–height relationship in the interferograms of non-deformed regions, such as the Linear model correction method [10] and the spatial power law method [11]. However, the relationship between the phase and the elevation in these methods is mostly linear, which is unable to estimate the variation in the nonlinear trend and cannot be applied to a large-scale area. (4) Atmospheric correction methods combining external data. For example, the newly released ERA-5 atmospheric reanalysis dataset (ERA5) from the European Centre for Medium-Range Weather Forecasts (ECMWF) [12] and the global coverage, near-real-time Generic Atmospheric Correction Online Service for InSAR (GACOS), which combines GPS observations with high-resolution ECMWF data [13]. They can provide complete information on tropospheric delays, but such methods are usually limited by the spatial and temporal resolution of the interferograms and external data and require a dense number of atmospheric observation sites in the region.

Due to the spatial and temporal variations in the atmospheric refractive index, the stratified tropospheric delay usually exhibits seasonal oscillations [14,15]. The spatial–temporal filtering method assumes that the atmosphere is not related in time and only has a weakening effect on the turbulently mixed delay that changes randomly in time and space, and it cannot weaken the error effect of a vertically stratified delay [16]. Therefore, it is not suitable for most areas where vertically stratified delays dominate. Secondly, the method of estimating tropospheric delay by GNSS observation is easily limited by the number of GNSS stations, and the spatial resolution is low in the application of tropospheric delay correction, which is not applicable in most areas. So, accordingly, it is of little significance to evaluate these two methods. The empirical linear model correction method and the method combining external data, as the methods commonly used today, can predict the tropospheric delay with different degrees of seasonal fluctuations in different geographic regions. However, they also have their own limitations, which are different for identifying the variations of atmospheric delay signals caused by the spatial–temporal factors in the InSAR. The empirical linear model method is based on the relationship between the phase and the elevation in the interferogram, and it is only dominant in estimating the vertically stratified delay component. The GACOS method calculates the zenith tropospheric delay (ZTD) through low-temporal-resolution meteorological data, and the estimation results of the turbulently mixed delay component are poor, and the stability is low. Atmospheric reanalysis information from ERA5 has the highest temporal resolution, and the ERA5 method effectively removes seasonal atmospheric influences from each interferogram, but turbulent mixing delays contained in the residual delay phases are not removed.

The above tropospheric delay correction methods are constrained by a number of factors, and different correction methods have different sensitivities to vertically stratified delays and turbulently mixed delays. It is difficult to effectively weaken the influence of the tropospheric delay on InSAR measurement deformation results at any region and time. Therefore, there is no method that can be used as a general tropospheric delay correction method under different geographical conditions. It is necessary to combine terrain, season, and meteorological factors to determine the appropriate tropospheric delay correction method. This study took an alpine gorge area covered with various terrains in the Dali Bai Autonomous Prefecture in southwestern China, which is significantly affected by monsoon climate, as an example. Based on the small baseline subset (SBAS) technique [17,18], the Sentinel-1A image data is used for time-series InSAR processing. Considering the influence of temporal and spatial variation, we quantitatively evaluated the effects of the empirical linear model, the GACOS, and the ERA5 delayed tropospheric correction methods in the region during different seasons and in different terrains.

## 2. Materials and Methods

### 2.1. Tropospheric Delay Correction

The InSAR tropospheric effect is caused by spatial and temporal variations in the atmospheric refractive index between two SAR observations related to air pressure, air temperature, and water vapor. The generated tropospheric delay phase consists of hydrostatic, wet liquid water and ionospheric delays. The liquid water delay caused by the refraction of water droplets in clouds and the ionospheric delay generated by the ionospheric effect have little effect on C-band SAR satellites [19,20]. Therefore, the atmospheric refractive index N can be decomposed into two parts: the hydrostatic refractive component, $N_{hydro},$ and the wet component, $N_{wet}$, which can be calculated by Equation (1). Meanwhile, Equation (2) is utilized to integrate the atmospheric refractive index, N, along the radar line-of-sight (LOS) direction between the altitude $z=z_{1}$ and the reference altitude value of the top of the troposphere, $z_{ref}$, to obtain the corresponding tropospheric delay phase, $\varphi_{tropo}$:

(1) $$N=N_{hydro}+N_{wet}=k_{1}\frac{P}{T}+k_{2}\frac{e}{T}+k_{3}\frac{e}{T^{2}}$$

(2) $$\varphi_{tropo}=\frac{-4\pi }{\lambda }\cdot \frac{10^{-6}}{\cos\theta }\int_{z_{1}}^{z_{ref}}\left(N_{hydro}+N_{wet}\right)dz$$

where P represents the total atmospheric pressure (hPa); T represents the temperature (K); e refers to the partial pressure of the water vapor (hPa); and the values of the empirical constants $k_{1}$, $k_{2}$, and $k_{3}$ are taken to be 0.776, 0.704, and 3.75 × 10^3^ K^2^·Pa^-1^, respectively. The parameter λ is the radar wavelength; θ is the radar angle of incidence; and the value of $z_{ref}$ is 15 km, beyond which altitude the effect of elevation on the atmospheric refractive index can be disregarded.

Notably, the tropospheric delay phase at each pixel in the interferogram represents a relative change, and the value of the tropospheric delay phase, $\varphi_{tropo},$ can be expressed as the difference in the tropospheric delay phase, $∆\varphi_{tropo},$ between the reference image and the auxiliary image as they are imaged:

(3) $$∆\varphi_{tropo}=\varphi_{s}-\varphi_{r}$$

where φ denotes the phase observation and the subscripts r and s denote the reference and auxiliary images, respectively.

#### 2.1.1. Empirical Linear Model Correction Method

The tropospheric delay has a strong correlation with the observed topography. By fitting the phase and elevation of the tropospheric delay in the InSAR non-deformed region, the Linear method can obtain a model of the phase as a function of elevation for estimating the vertically stratified delay component of the tropospheric delay due to the topographic relief. The empirical linear modeling approach has strong stability in estimating the delay related to terrain, and the difference between the reference and auxiliary images corresponding to the tropospheric delay signal, $∆\varphi_{tropo},$ can be linearly expressed as

(4) $$∆\varphi_{tropo}=\Delta \varphi_{0}+Kh$$

where $\Delta \varphi_{0}$ is the constant phase offset in the interferogram; h is the elevation; and K is the correlation coefficient between the tropospheric delay phase and the elevation.

In the Linear model, estimating the correlation coefficient K is the key step. The wavelet transform theory is utilized to divide the terrain and interferograms into a number of different spatial scales, where some specific scales show high terrain–phase correlation, and the resulting K values are relatively stable compared with other scales. The K estimates obtained by this method can ignore the effects of other phase components such as orbital errors and tectonic deformation, but only if the interferogram scales are consistent; otherwise, the estimated model parameters are not the same, which will lead to bias in the tropospheric delay estimation.

The Linear method does not distinguish well between the deformation phase and the tropospheric delay phase, so regions where significant deformation occurs need to be separated in the analysis.

#### 2.1.2. GACOS Correction Method

GACOS extends the Iterative Tropospheric Decomposition (ITD) model by incorporating the global atmospheric analysis dataset, published by the ECMWF, to achieve spatial resolutions up to 0.1° and temporal resolutions up to 6 h. The GACOS correction method, driven by this high-resolution forecast (HRES) model, allows the generation of time-continuous and highly accurate troposphere-corrected interferograms by separating the vertical stratification delay and turbulent mixing delay from the tropospheric zenith total delay (ZTD) by combining GNSS observations [21]. The ZTD at position k is defined as

(5) $${ZTD}_{k}=T_{\left(x_{k}\right)}+L_{0}e^{-\beta {\bar{h}}_{k}}+\varepsilon_{k}$$

where T is the turbulent delay component; $x_{k}$ is the station coordinate vector in the local topographic coordinate system; and $L_{0}$ is the vertically stratified component at sea level. An exponential function with a coefficient of β is used to represent the stratified delay component; ${\bar{h}}_{k}=(h_{k}-h_{min})/(h_{max}-h_{min})$ denotes the altitude scale; and $\varepsilon_{k}$ denotes the residuals, i.e., the residual unmodeled stratified delay signals and turbulent delay signals.

The model for calculating the stratified delay component S can also be expressed as

(6) $$S_{i}=L_{0}e^{-\beta h}=>\left\{\begin{array}{c} S_{m}^{G}=L_{0}e^{-\beta h_{m}}\\ S_{n}^{E}=L_{0}e^{-\beta h_{n}} \end{array}\right.,\;P_{i}=\left[\begin{array}{cc} P_{G} & 0\\ 0 & P_{E} \end{array}\right]$$

where $S_{m}^{G}$ and $S_{n}^{E}$ represent the ZTD of the GNSS at position m and the ZTD of the ECMWF at position n (referred to as GNSS-ZTD and ECMWF-ZTD), respectively. The weight matrix $P_{i}$ is determined according to the quality of the GNSS-ZTD and the ECMWF-ZTD. The GNSS-ZTD captures the temporal variability of the troposphere better than the ECMWF-ZTD, while the higher spatial resolution and uniformly distributed stations of the ECMWF make it superior to the GNSS for interpolation.

The model for calculating the turbulent delay components T can be expressed as

(7) $$T_{ii}=\sum_{i=1}^{k}w_{ui}T\left(x_{i}\right),\;w_{ui}=\frac{p_{i}d_{ui}^{-2}}{\sum_{i=1}^{k}p_{i}d_{ui}^{-2}}$$

where u and i are the indexes of the target and reference positions, respectively. $w_{ui}$ is the weight assigned to each turbulence delay at the target position, which is determined by the weight matrix, $P_{i},$ of the GNSS-ZTD and the ECMWF-ZTD and the horizontal distance, $d_{ui},$ from the target position to the reference position.

The ITD model first assumes that there is no turbulent delay signal and derives a pair of fixed $L_{0}$ and β parameters through Equation (5); it then calculates the turbulent delay of each reference station based on the residuals $\varepsilon_{k}$ through the inverse distance weighting (IDW) function and subtracts this turbulence component from the ZTD of each reference station to generate a new set of exponential coefficients, and it outputs the stable exponential coefficients by repeating the iterations. Finally, using its resulting set of $L_{0}$ and β parameters that converge in the region, plus the turbulence delay component and the residuals of each GPS station, these are interpolated to obtain the ZTD value of each station, and the finalized $L_{0}$ and β coefficients values are added to $L_{0}e^{-\beta {\bar{h}}_{k}}$ to compute the stratified delay component, and the two are summed up to produce a relative ZTD value [22].

In addition, for the InSAR data, the signal propagation path is the radar line of sight (LOS), and the zenith tropospheric delay (ZTD) can be converted to the radar line of sight by the projection function:

(8) $$\Delta L^{LOS}=\frac{1}{\cos\theta }∆L^{Z}$$

where $\Delta L^{LOS}$ and $∆L^{Z}$ represent the delay of the radar line of sight and the delay of the zenith direction, respectively. The parameter $\frac{1}{\cos\theta }$ is the projection transformation function, and θ is the incidence angle of the radar signal.

#### 2.1.3. ERA5 Dataset Correction Method

ERA5 covers the meteorological reanalysis datasets from 1950 to the present with high temporal resolution (1 h) and spatial resolution (0.25°). In the case that the meteorological reanalysis data of ERA5 and the acquisition time of SAR image data are not completely synchronized, four variables, including air temperature, potential, specific humidity, and barometric pressure values, are extracted from the pressure layers closest to the acquisition time of the SAR images, assuming that the atmospheric parameters of the two adjacent moments are linearly varying [23]. The reanalysis parameters of each layer are interpolated to the grounding point using a bilinear interpolation method to calculate the ZTD and to compensate for the height difference between the grounding point and the atmosphere layers [24]. Then the hydrostatic delay and the wet delay of the main image and the auxiliary image are calculated respectively. The atmospheric delay of each pixel in the SAR image at time t can be calculated by integrating the elevation z:

(9) $$\varphi_{tropo}^{hydr}\left(z,t\right)=\frac{-4\pi }{\lambda }\cdot \frac{10^{-6}}{\cos\theta }\cdot \frac{k_{1}R_{d}}{g_{m}}\left[P_{\left(z,t\right)}-P_{\left(z_{0},t\right)}\right]$$

(10) $$\varphi_{tropo}^{wet}\left(z,t\right)=\frac{-4\pi }{\lambda }\cdot \frac{10^{-6}}{\cos\theta }\int_{z}^{z_{ref}}\left[k_{2}\frac{e\left(z,t\right)}{T\left(z,t\right)}+k_{3}\frac{e\left(z,t\right)}{T{\left(z,t\right)}^{2}}\right]dz$$

where $R_{d}$ denotes the dry-air specific gas constant. $R_{d}=287.05\;J\cdot {kg}^{-1}\cdot K^{-1}$. $g_{m}$ is the acceleration of gravity, and $g_{m}=9.8\;m\cdot s^{-2}$.

In this study, the partial pressure of the water vapor, e, is needed to calculate the wet delay, and the relevant meteorological parameter extracted from the ERA5 reanalysis data is the relative humidity, $R_{e}$. We need to convert $R_{e}$ into e by using the Clausius–Clapeyron equation [25]:

(11) $$e\left(z\right)=e^{*}(z)\frac{R_{e}(z)}{100}$$

(12) e*z=ew*z=a1ea3wTz − T0Tz − a4w ,          Tz≥T0ei*z=a1ea3iTz − T0Tz − a4i ,        Tz≤Ti                                 ei*z+ew*z−ei*zTz − Ti2T0 − Ti ,       T0<T(z)≤Ti

where $e^{*}(z)$ denotes the partial pressure of saturated water vapor, $e_{w}^{*}\left(z\right)$ denotes the partial pressure of supersaturated liquid water, and $e_{i}^{*}\left(z\right)$ denotes the supersaturated ice partial pressure. $T_{0}=273.16\;K$, $T_{i}=250.16\;K$, $a_{1}=611.21\;hPa$, $a_{3w}=17.502$, $a_{4w}=32.19\;K$, $a_{3i}=22.587$, and $a_{4i}=-0.7\;K$.

Considering that the key to observing the delayed phase of the troposphere is to match the image acquisition time and region, the ZTD can be derived as

(13) $$ZTD={ZTD}_{up}+{ZTD}_{do}$$

where ${ZTD}_{up}$ and ${ZTD}_{do}$ are the upper and lower parts of the reanalyzed top layer, respectively. Considering that the tropospheric delay above the observation boundary of the ERA5 dataset can be disregarded, the tropospheric delay phase of each pixel can be obtained by projecting the ZTD in the radar line-of-sight direction according to Equations (1)–(3) in combination with the radar incidence angle and the DEM at the grounding point below the boundary.

### 2.2. Study Area and Data Processing

#### 2.2.1. Overview of the Study Area

**Figure 1:**
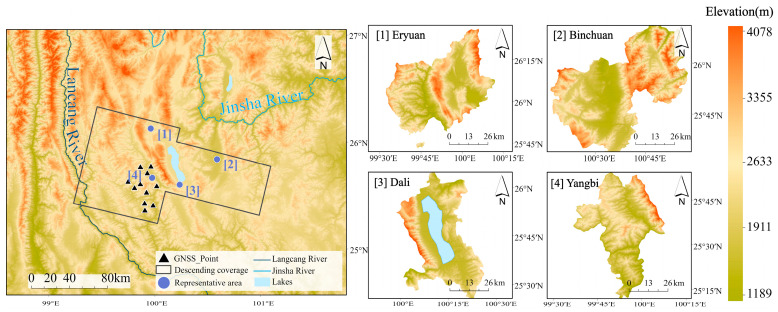
Topographic map of the study area. The representative area ID and name are labeled in each subplot.

As shown in Figure 1, we chose the junction area of the Hengduan Mountains and the Yunnan–Kweichow Plateau as the study area (99°24′–100°48′ E, 25°18′–26°18′ N), which contains different topographic regions such as alpine valleys, steep slopes of the middle mountains, plains, basins, river valleys, etc. The terrain shows a trend of being high in the northwest and low in the southeast, with a minimum elevation of 1150 m and a maximum elevation of 4112 m, and the average elevation was 2162 m. Due to the collision and extrusion of the Eurasian plate and the Indian Ocean plate, a plateau topography was formed in the region. With this process, the fold mountain system was formed, and the topography was undulating. Under the joint influence of the planetary wind belt and the huge topography, the region is significantly affected by the subtropical plateau monsoon climate, with significant non-periodic changes in weather and seasonal variations in precipitation, including the northern subtropical, central subtropical, southern subtropical, and alpine-mountainous climate-type zones. Based on the characteristics of the spatial and temporal variability of water vapor content influenced by topography and seasonal factors, we selected four representative regions located in the north, east, south, and west of the study area for study and analysis; namely, Eryuan (99°48′–100°06′ E, 25°48′–26°06′ N), Binchuan (100°24′–100°42′ E, 25°36′–25°51′ N), Dali (100°12′–100°24′ E, 25°33′–25°45′ N), and Yangbi (99°51′–100°03′ E, 25°30′–25°42′ N). The direction of the plateau monsoon circulation in the four regions is completely consistent with the direction of the monsoon formed by the difference in the thermal properties of the sea and land. They are typical areas for studying the tropospheric delay in the plateau monsoon climate zone. The Eryuan region mainly belongs to the climatic type of the northern subtropical plateau, and part of its territory is an alpine-mountain climatic zone. The western part of the region is dominated by high mountain valleys, while the eastern part is a basin, which makes the regional microclimate characteristics obvious. The terrain types in the Binchuan area are mainly fault basins between mountains, and the three-dimensional climate is obvious. The terrain of the Dali area is flat, dominated by alluvial plains and hills. The Yangbi area spans the Hengduan Mountain alpine gorge area, with an average height difference of more than 1500 m between the mountain valleys and the ridge, and the terrain is extremely undulating.

#### 2.2.2. Data Sources

In this study, 25 C-band scenes and Sentinel-1A interferometric wide (IW)-swath imaging mode descending-track data of single-look complex (SLC) images were collected from April 2021 to April 2022. The imaging time of the descending tracks SAR image data was 23:14 coordinated universal time (UTC). The topographic phase component was removed using the NASA Shuttle Radar Topography Mission (SRTM) external DEM data with a spatial resolution of 30 m that was used to remove the topographic phase component; GACOS zenith tropospheric delay (ZTD) product data were obtained from the website https://www.gacos.net (accessed on 12 October 2022). The ERA5 dataset for the atmospheric reanalysis product was obtained from the website https://cds.climate.copernicus.eu (accessed on 8 November 2022). Basic information on the SAR images is presented in Table 1.

#### 2.2.3. Data Processing

**Figure 2:**
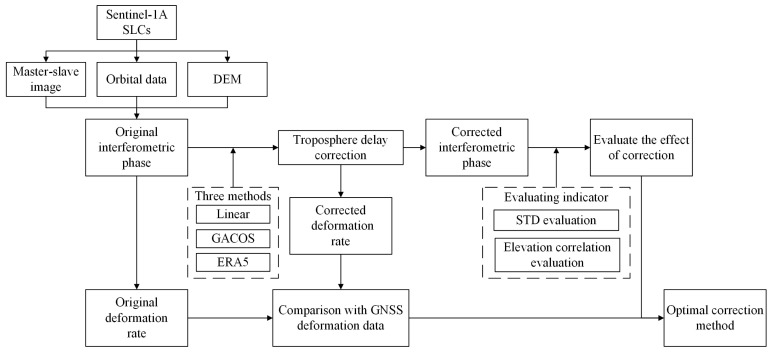
Technical flow chart of evaluating and estimating the three tropospheric delay correction methods.

An image from 13 October 2021, was selected as the primary image, and the remaining 24 views were secondary images. First, the original interferometric phase was obtained by preprocessing the raw SLC data using the InSAR Scientific Computing Environment (ISCE v2.6.1) software [26]. Second, a time-series InSAR analysis was performed based on the SBAS technique and the Stanford method for persistent scatterers (StaMPS) method [27,28]. The maximum temporal baseline threshold was set to 120 d and the maximum spatial baseline threshold was 180 m during the processing, 20 × 5 multi-views were applied to the interferograms in the distance and azimuth directions to enhance the signal-to-noise ratio, respectively, and a total of 92 descending-track interference pairs were obtained. The statistical-cost network flow algorithm for phase unwrapping (SNAPHU v2.0.5) software was then used to obtain the time-series surface deformation information [29]. After removing DEM errors, orbital errors, and other systematic errors from the interferograms, the deformation phase in the LOS direction of the SAR satellites was obtained by correcting the tropospheric delay of the InSAR using the Linear, GACOS, and ERA5 methods. Finally, the phase standard deviation (STD) and phase–elevation correlation were introduced to analyze and evaluate the effect of the tropospheric delay correction. Meanwhile, the cumulative surface displacement data from the GNSS surface deformation monitoring stations were taken as the true values and compared and analyzed with the InSAR deformation monitoring results after the delayed tropospheric correction. Based on the above, the performance of the three tropospheric delay correction methods was evaluated by combining factors such as topography, season, and meteorological conditions to determine the most suitable tropospheric delay correction method for the region. The technical process is illustrated in Figure 2.

## 3. Results

### 3.1. Methods of Analysis and Assessment

In this study, three methods, Linear, GACOS, and ERA5, were used to correct interferograms from spring (16 April 2021–10 May 2021), summer (15 June 2021–9 July 2021), fall (13 October 2021–6 November 2021), and winter (12 December 2021–5 January 2022). The coverage of the study area for the interferograms was corrected for tropospheric delays. Regions 1–4 showed different tropospheric delay corrections for different seasonal segments. Based on the different topographic and meteorological conditions in each of the four regions, we present and discuss four representative examples of corrections using the Linear, GACOS, and ERA5 methods and show the statistical and analytical results for each of the four regions grouped by season.

There are deformation displacements caused by various factors in each region every year, and small deformation phases cannot be accurately calculated. For this study, we could not know in advance the amount of deformation displacement in the whole study area, and it was difficult to completely separate the deformation phase. We eliminated the deformation phase as much as possible to reduce the influence of the deformation phase. According to the standard released by the China Geological Disaster Prevention Engineering Association, the area with an absolute value of annual deformation rate of less than 5 mm/y in the LOS direction obtained based on SBAS-InSAR technology is regarded as a non-deformation area [30]. The deformation signals in these regions with a short time baseline are extremely weak, and the tropospheric delay phase has the largest magnitude among the remaining speckle noise phases. We analyze and evaluate the correction effects of the three tropospheric delay correction methods by filtering out the phase points of the non-deformed region in the interferogram with a time baseline of 24 days and calculating their phase values [31].

#### 3.1.1. Evaluation of the Phase Standard Deviation

The phase value of any pixel on the InSAR interferogram is the phase difference between the corresponding pixels of the main and auxiliary images. The reference ellipsoid phase, terrain phase, deformation phase, and atmospheric phase account for the main part. After removing residuals such as orbital and DEM errors by the time-series InSAR technique, we assume that no deformation occurs on the surface, and then only the atmospheric phase is included in the remaining phase values. Therefore, we introduce the phase standard deviation (STD) metric to evaluate the correction quality of the three methods for tropospheric delay correction, which is evaluated by the following formula:

(14) $$\sigma =\sqrt{\frac{1}{N}\sum_{i-1}^{N}{\left(x_{i}-\mu \right)}^{2}},\mu =\frac{1}{N}\sum_{i=1}^{N}x_{i}$$

where N represents the number of phase points obtained in the time-series InSAR technology after screening, $x_{i}$ denotes the phase value of the corresponding phase point, and μ denotes the average phase value.

In this study, the phase standard deviation, σ, of each interferogram is calculated by screening out the non-deformed region and counting its interference phase value. A larger value of STD in the non-deformed region indicates a more significant tropospheric delay signal in that region [32].

#### 3.1.2. Phase–Elevation Correlation Analysis Based on the Pearson Correlation Coefficient

In the process of removing the tropospheric delay using time-series processing, the effect of turbulence delay is weakened while the vertical stratification delay dominates, and the interferogram phase shows a strong correlation with elevation [33]. Tropospheric delay is closely related to elevational changes, and this study measures the ability of the three methods to weaken the vertical stratification delay by analyzing the relationship between phase and elevation and calculating their Pearson correlation coefficients:

(15) $$r=\frac{n\sum_{i=1}^{n}x_{i}y_{i}-\left(\sum_{i=1}^{n}x_{i}\right)\left(\sum_{i=1}^{n}y_{i}\right)}{\sqrt{n\sum_{i=1}^{n}x_{i}^{2}-{\left(\sum_{i=1}^{n}x_{i}\right)}^{2}}\cdot \sqrt{n\sum_{i=1}^{n}y_{i}^{2}-{\left(\sum_{i=1}^{n}y_{i}\right)}^{2}}}$$

where n represents the number of phase points in the region, and x and y represent two sets of data on the interferometric phase value and elevation, respectively.

It is worth noting that the original interferogram generated by SBAS-InSAR time series processing contains both the tropospheric delay signal and the deformation signal. The tropospheric delay signal and the elevation show an obvious linear relationship, but the deformation signal causes the unwrapped phase in some areas to be discontinuous, which will mask the tropospheric delay signal. Therefore, as in Section 3.1.1, we separate the deformation signal by excluding phase values in the deformation regions, which, in turn, provides a more accurate assessment of the effectiveness of the three methods in attenuating the tropospheric delay.

### 3.2. Representative Case Study

#### 3.2.1. Binchuan and Eryuan in Spring

**Figure 3:**
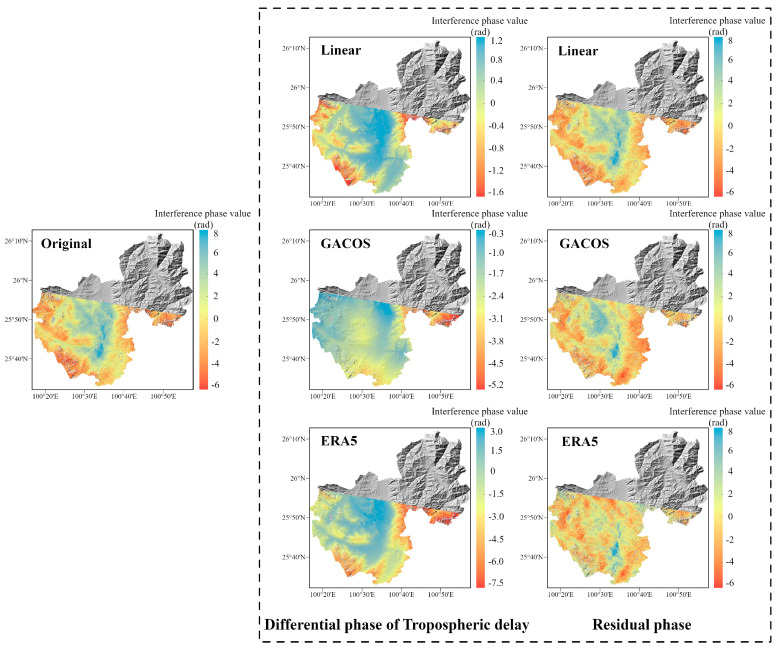
The correction effect of the three tropospheric delay correction methods on InSAR interferograms in the Binchuan area. The first column is the original interferogram, the second column is the differential phase of the tropospheric delay estimated by the three methods, and the third column is the interferogram after tropospheric delay correction.

**Figure 4:**
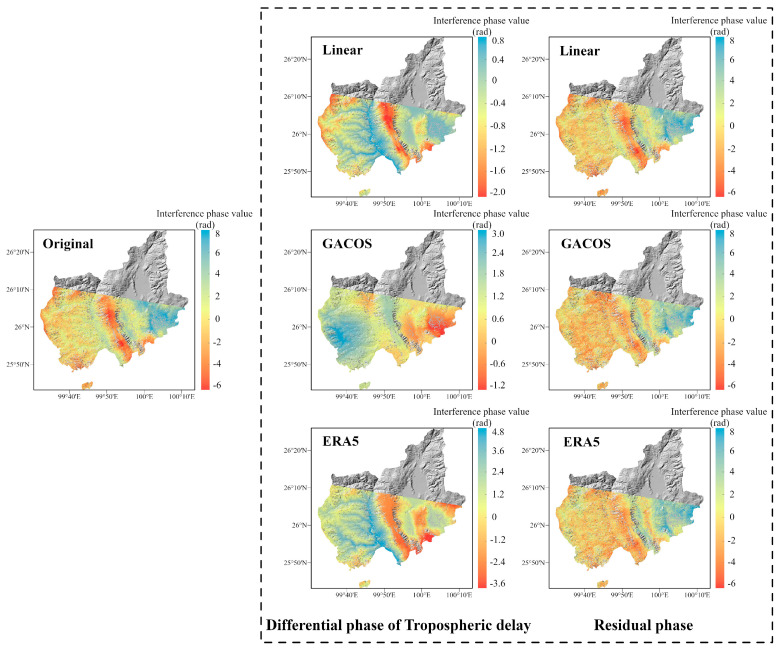
The correction effect of the three tropospheric delay correction methods on InSAR interferograms in the Eryuan area. The presentation form is the same as for the Binchuan area in Figure 3.

In spring, the interferograms are banded with a significant dependence on topography, especially in the Binchuan area located on the south bank of the Jinsha River and the Eryuan area located in the Hengduan Mountains region (shown in Figure 3 and Figure 4). Both regions are significantly affected by the monsoon climate under this seasonal period, with unstable meteorological conditions and some turbulent mixing movements of the atmosphere in the territory. The randomness of physical properties such as pressure, velocity, and temperature at each point of turbulence is very large, which leads to the heterogeneity of the atmospheric refractive index in three-dimensional space, resulting in irregular and small-scale tropospheric delay changes in space. As shown in the differential phase of the tropospheric delay results in Figure 3, the atmospheric delay phase estimated by the GACOS model is relatively divergent. We speculate that the reason for this phenomenon is that heavy rainfall occurred in the Binchuan area during the time period of SAR image acquisition, and the GACOS method, because of the large number of weather models it combines with, overestimates or underestimates the atmospheric delays occurring in certain atmospherically unstable regions. Both the Linear method and the ERA5 method estimate terrain-related delays. However, the estimated delay phase values show that ERA5 is more capable of recognizing the vertical stratification component. The final residual phase results show that the ERA5 correction is effective, while the correction effects of the Linear and GACOS methods need to be further analyzed quantitatively for comparison.

As shown in Figure 4, the tropospheric delayed phase values estimated by the Linear and ERA5 methods show a clear divergence in the eastern part of Eryuan. This indicates that the eastern part of Eryuan in the interferogram represents a small-scale irregular atmospheric signal, and the localized turbulent component dominates in this region. The divergence of the tropospheric delay phase estimated by the GACOS method in this region is small, indicating that it can identify some turbulence changes. However, GACOS fails to estimate a more accurate terrain-related delay in the western part of Eryuan, while, in contrast, the other two methods do so. From the residual phase results, owing to the spatial and temporal randomness of the turbulent mixing delay, none of the three tropospheric delay correction methods can accurately identify or attenuate this error.

**Figure 5:**
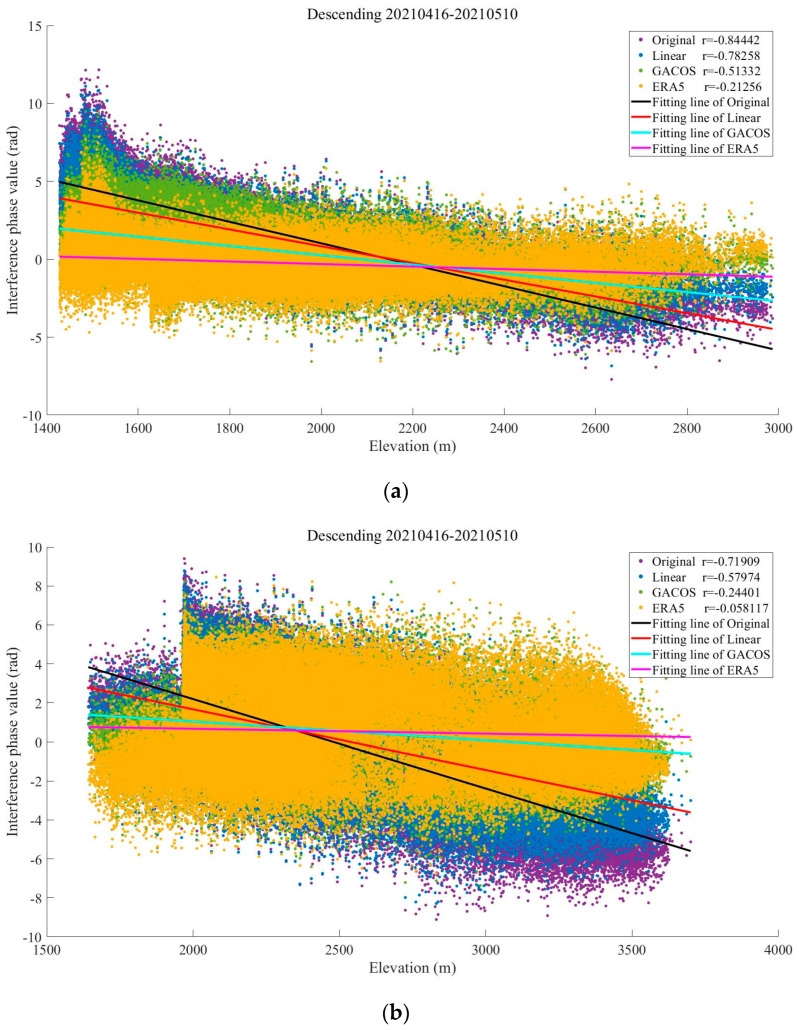
The relationship between the interferometric phase and elevation: (**a**) Binchuan area; (**b**) Eryuan area.

As shown in Figure 5, the interferogram phases and elevations were negatively correlated in both regions before the tropospheric delay correction, a phenomenon that may be due to the topographic relief or solar radiation variations caused by quasi-stationary frontal weather systems [34]. The absolute value of the Pearson correlation coefficient between the original phase and the elevation of the interferogram in Binchuan area is as high as 0.84, and the atmospheric delay phase is strongly correlated with the terrain. The absolute value decreased after the tropospheric delay correction using the three methods, indicating that the vertical stratification delay was partially mitigated. The correlation coefficient between the phase and elevation in the Eryuan region is slightly smaller than the former one, which is 0.72. This may be due to the barrier of the Hengduan Mountains, which led to the formation of a frontal system in the region in spring, and more divergent phase values were generated due to different meteorological conditions in the east and the west. Among them, the ERA5 method provides the best improvement in both conditions, and the absolute values of the coefficients of the original interferograms corrected by it decrease to 0.21 and 0.06, which alleviate the stratified residuals to a large extent.

**Figure 6:**
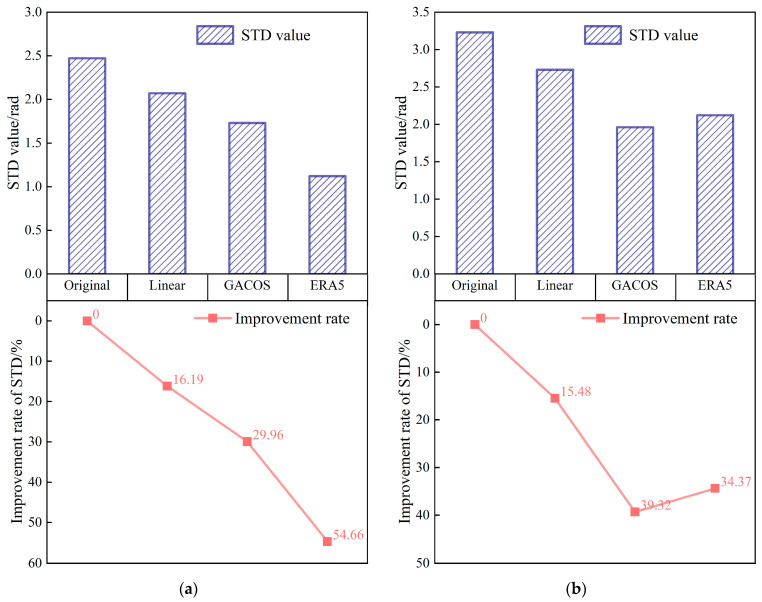
Statistical figure of interference phase standard deviation: the histogram represents the standard deviation, and the line chart represents the standard deviation improvement rate. (**a**) Denotes the Binchuan area. (**b**) Denotes the Eryuan area.

The Binchuan region contains basins and discrete mountain ranges, and the STD of the original interferogram in spring is 2.47 rad, which is reduced to 2.07 rad, 1.73 rad, and 1.12 rad after correction using the three methods, with improvement rates of 16.19%, 29.96%, and 54.66%, respectively (shown in Figure 6a). The Eryuan region has a complex topography with large undulations and is bounded by the Hengduan Mountains in the center, alpine gorges in the west, and valley basins in the east. The STD before interferogram correction in this area is as high as 3.23 rad in spring, which decreases to 2.73 rad, 1.96 rad, and 2.12 rad after correction using the tropospheric delay correction methods based on the Linear, GACOS, and ERA5 methods, respectively, which represent improvement rates of 15.48%, 39.32%, and 34.36% (shown in Figure 6b).

The results show that all three methods effectively mitigate the tropospheric delay in the original interferograms under different terrain conditions in the same season. However, the differences in meteorological conditions due to the different topographic distributions make the correction effects of the three methods different. The GACOS method, with a spatial resolution of 0.1°, can more accurately separate different delay signals under the same region, which gives it an advantage in Eryuan, where the influence of the frontal system is obvious, but produces errors due to overcorrection in Binchuan, where the whole region is controlled by the warm and humid spring airflow. On the contrary, the ERA5 method has strong stability for identifying tropospheric delay signals. Finally, the Linear method is less effective at estimating regions with small time and spatial scales.

#### 3.2.2. Yangbi in Winter

**Figure 7:**
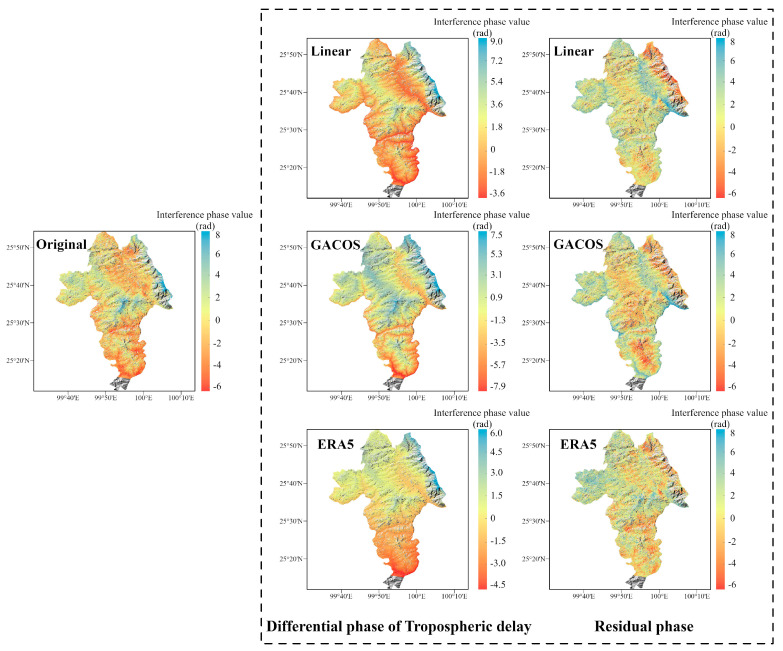
The correction effect of the three tropospheric delay correction methods on InSAR interferograms in the Yangbi area. The presentation form is the same as for the Binchuan area in Figure 3.

The Yangbi area belongs to the Hengduan Mountain alpine canyon area, which contains many ridge–valley combination terrains. The terrain is undulating, and the precipitation has vertical distribution characteristics. In winter, the region is under the influence of the southwest monsoon and under the control of a dry and warm western air mass. The water vapor content is the lowest in all seasons, and the peak precipitable water volume (PWV) is generally below 50 mm, with low daily variability [35]. Under the combination of these conditions, turbulence effects are weakened, and stratification effects dominate, as shown in the interferogram in Figure 7, which shows the stratification component related to the topography.

**Figure 8:**
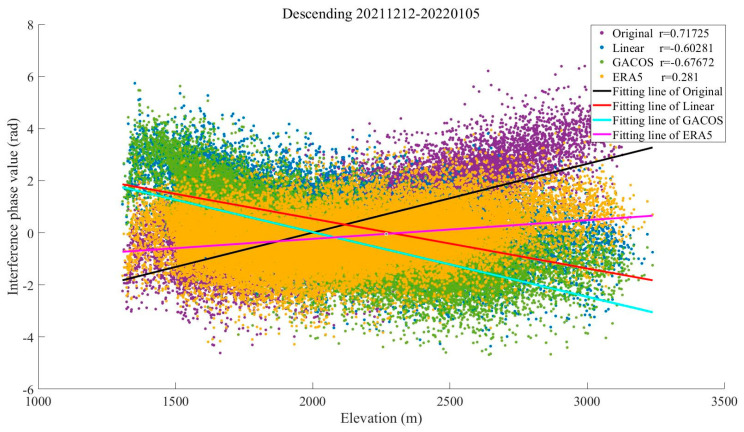
The relationship between the interferometric phase and elevation in the Yangbi area.

As can be seen in Figure 8, the Pearson correlation coefficient of the interferograms before correction is as high as 0.72. The results show that the ERA5 method obviously reduces the layered component in the interferograms, and the absolute value of the Pearson correlation coefficient decreases to 0.28. The correction effect of the Linear and GACOS methods on the vertical layered delay is not obvious in this case, and the absolute value of the Pearson correlation coefficient decreases to only 0.60 and 0.68. This may be due to the high frequency of earthquakes in the Yangbi area, with the most recent strong earthquake occurring in May 2021 and with many aftershocks since then. The GACOS method combined with the GNSS has a low estimation accuracy of the tropospheric delay in this area. The Linear method is not suitable for small-area analysis. Therefore, both of these are not as good as the ERA5 method in estimating the vertical stratification, even in the Yangbi area, which has a dry climate and a stable atmospheric environment in winter [36,37].

**Figure 9:**
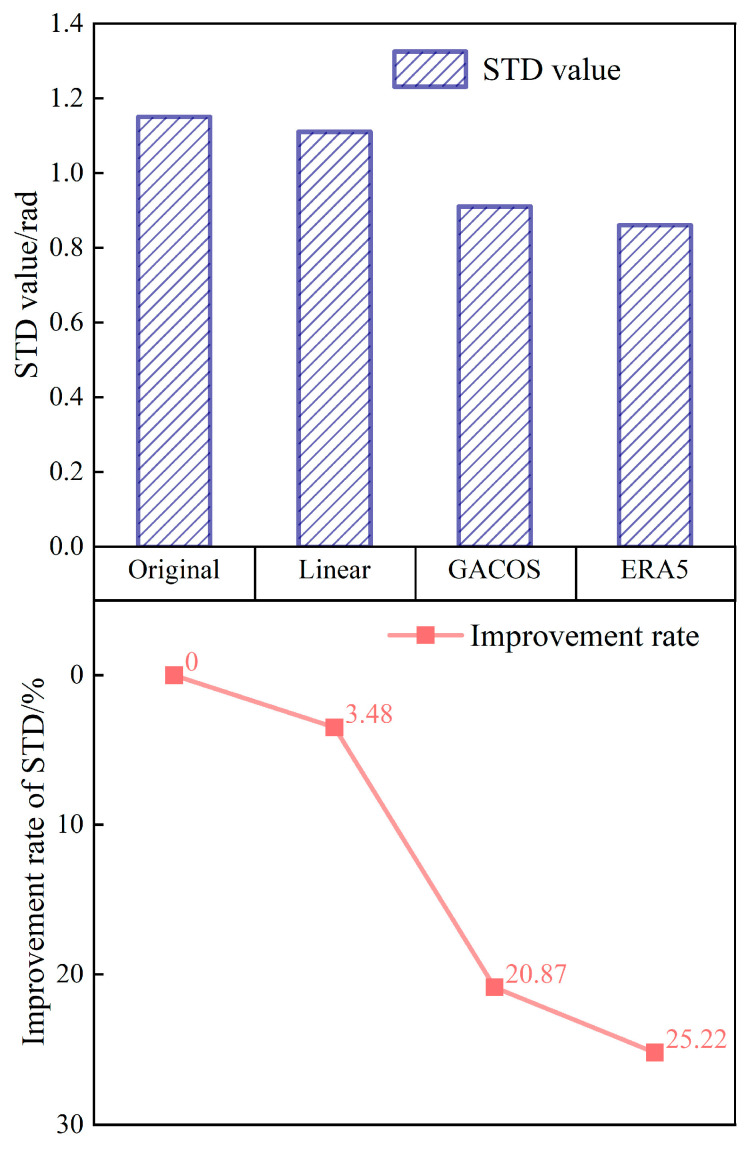
The phase standard deviation in the Yangbi area. The presentation form is the same as in Figure 6.

The STD of the interferograms before correction in Yangbi in winter was 1.15 rad, and the STD decreased to 1.11 rad, 0.91 rad, and 0.86 rad after correction using the three methods, with improvement rates of 3.47%, 20.87%, and 25.22%, respectively (shown in Figure 9). The ERA5 method showed the greatest reduction in STDs for this season and terrain, while the Linear method had a negligible corrective effect. The reason for the difference may be the limited extent and complex topography of the region, which make the ERA5 method, which combines atmospheric reanalysis data with higher spatial and temporal resolution, more capable of accurately identifying changes in the tropospheric delay phase based on the characteristics of the three-dimensional climate change. The results show that under the condition of dryness and less rain in winter, ERA5 shows great advantages for the ridge–valley combination area that has an obvious three-dimensional climate and frequent crustal activity.

#### 3.2.3. Dali in Summer

**Figure 10:**
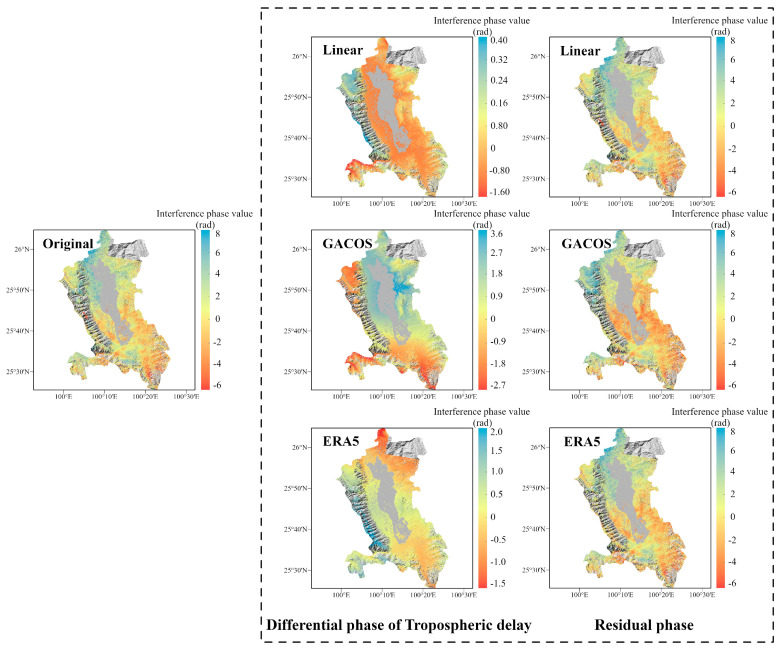
The correction effect of the three tropospheric delay correction methods on InSAR interferograms in the Dali area. The presentation form is the same as for the Binchuan area in Figure 3.

The Dali region is the transition zone between the Yunnan–Kweichow Plateau and the Erhai Lake Plain. Hills and low mountains are mainly distributed in the southeast, while the southern coastal area of Erhai Lake forms an alluvial plain due to long-term river alluvial flow, and the terrain is relatively flat. This causes the region to be affected by the southwest monsoon during the summer, when rainfall is high and irregularly distributed. From the interferogram result, Dali in summer shows a stronger trend of localized turbulence compared with the previous three regions (shown in Figure 10), which may be due to the abundant water vapor resources in the region and the short duration and high frequency of rainfall in this season. After correction using the three methods, the turbulence component was retained in the interferogram residual, and the nonlinear trend was more evident. However, the three methods are still able to estimate the weak tropospheric vertical stratification delay in the western mountainous and southeastern hilly areas of Dali. As can be seen from the tropospheric delay differential phase results, GACOS is more sensitive to topography in this case and is the most effective at recognizing topography-related delays.

**Figure 11:**
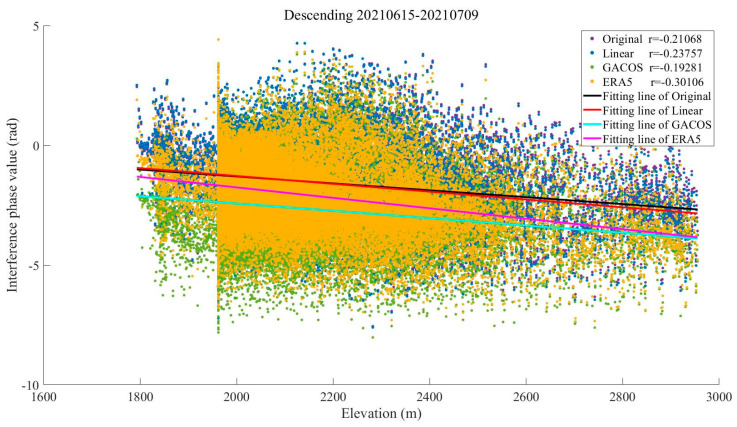
The relationship between the interferometric phase and elevation in the Dali area.

The closer the absolute value of the Pearson correlation coefficient between the phase and elevation of the original interferogram is to 1, the more obvious the vertical stratification effect is, and the closer it is to 0, the more obvious the turbulence effect is [38]. As shown in Figure 11, the absolute value of the correlation coefficient before correction in the Dali City area is 0.21, which shows more horizontal turbulence fluctuations than the areas described in Section 3.2.1 and Section 3.2.2. The absolute value of the correlation coefficient decreased to 0.19 after correction based on GACOS, indicating that the high-spatial-resolution GACOS product can identify the vertical stratification delay in some mountainous and hilly areas of the region. The scatter plots corrected by the Linear method and the ERA5 method are more dispersed, and the absolute values of the correlation coefficients increased to 0.24 and 0.30, respectively. All three methods are affected by the stronger turbulence tendency in the region, and all of them introduce additional disturbing noise in the interferograms.

**Figure 12:**
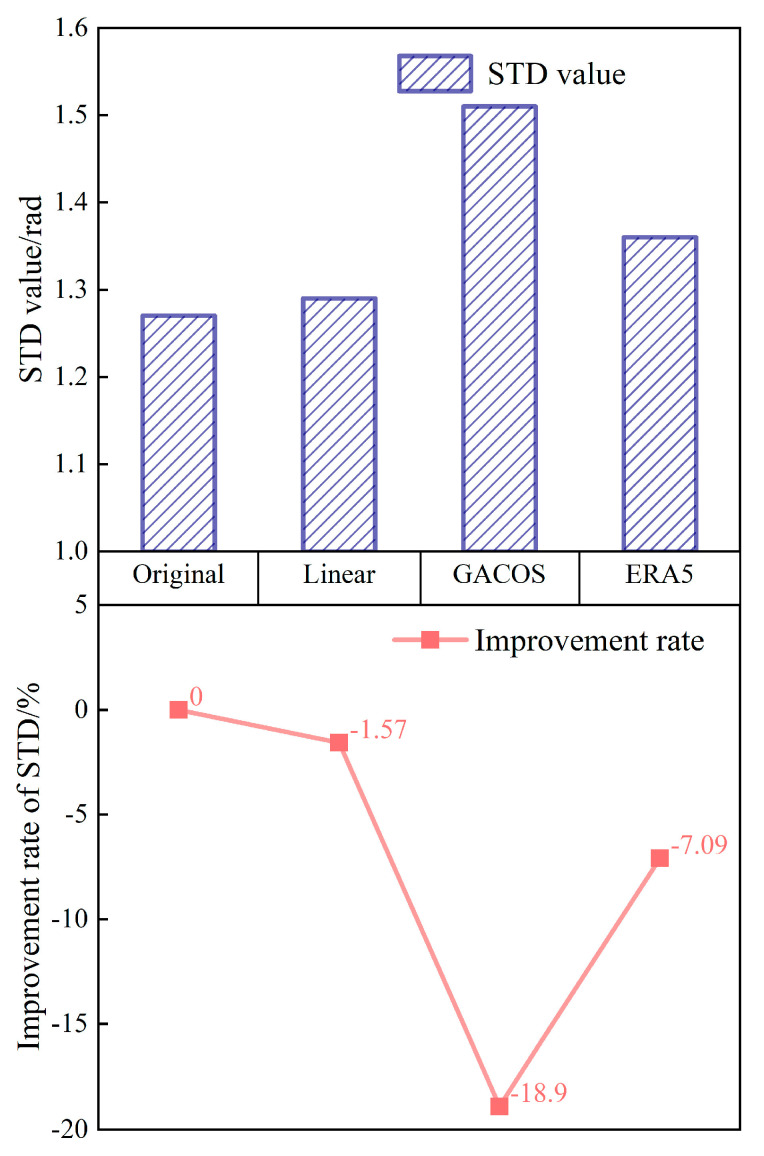
The phase standard deviation in the Dali area. The presentation form is the same as in Figure 6.

The image acquisition time is during summer when the humidity is highest and there are thunderstorms, which complicate the distribution and changes in atmospheric water vapor. In summer, the STD of the interferogram in southern Dali was 1.27 rad before correction and increased to 1.29 rad, 1.51 rad, and 1.36 rad after correction by the three methods. This indicates that the three tropospheric delay correction methods were not effective. The STD growth rates of the Linear, GACOS, and ERA5 methods were 1.57%, 18.90%, and 7.09%, respectively. It was difficult for all three methods to capture the small-scale local turbulence during the summer, and additional errors were introduced (Figure 12).

### 3.3. Comparison of GNSS and InSAR Deformation Results

**Figure 13:**
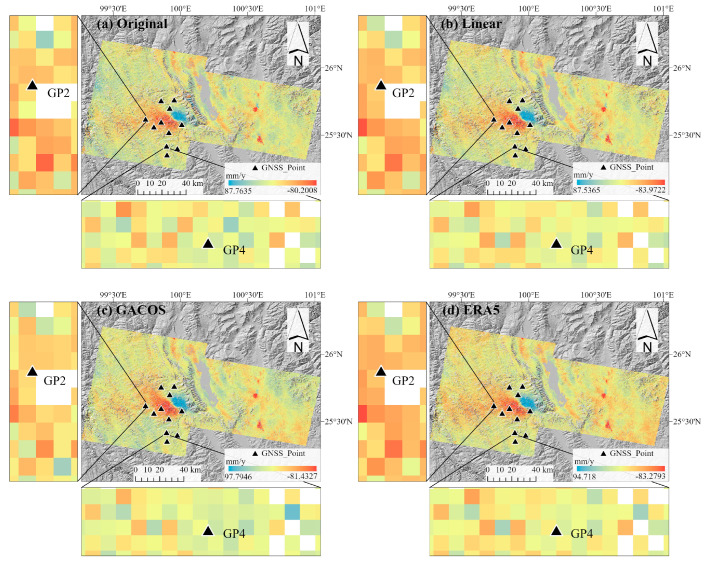
The results of the deformation rate change before and after tropospheric delay correction: (**a**) represents the original deformation rate diagram; (**b**–**d**) represent the deformation rate diagrams corrected by the three methods.

Based on the tropospheric delay correction results for the four areas in the east–west and north–south directions of the study area, the comprehensive correction effect of the ERA5 was the best. To evaluate the applicability of the three methods in areas greatly affected by the monsoons, nine GNSS stations were selected in the study area, and their deformation monitoring results projected in the LOS direction were compared with the deformation results before and after tropospheric delay correction [39]. The site distribution is shown in Figure 13, which shows the deformation rate results in the LOS direction of the study area before and after the tropospheric delay correction obtained by the SBAS-InSAR method. The results show that the deformation trend at most sites in the study area was relatively gentle over the year. The deformation rate signal displayed in the area with a stable geological structure was mainly caused by the tropospheric delay and shows a number of noise points and unsmooth noise. After the tropospheric delay correction, the nonlinear trend of the deformation rate in the non-deformed region was alleviated, eliminating these noise points and effectively smoothing the noise. The GNSS station we selected is located in an area with fault activity, and the study area experienced earthquakes during the GNSS and InSAR monitoring periods, causing clear deformation characteristics and making it difficult to distinguish the tropospheric delay signal. Therefore, further quantitative analysis is required here.

The nine GNSS stations selected in this study monitored the surface displacement in the region from 10 May to 30 November 2021. We projected the observations of the north–south, east–west, and vertical directions of the GNSS site to the radar line of sight of the SAR satellite system through the azimuth and incident angle information provided by the satellite platform and obtained the displacement of the GNSS in the LOS direction. Taking the monitoring results of each station on 10 May 2021, as the reference value, the cumulative surface displacement of each time node relative to the reference point was calculated. We used the data results as the true values, compared them with the deformation results obtained by the time-series InSAR before and after the tropospheric delay correction, and calculated the root-mean-square error (RMSE) of the four methods relative to the true value.

**Figure 14:**
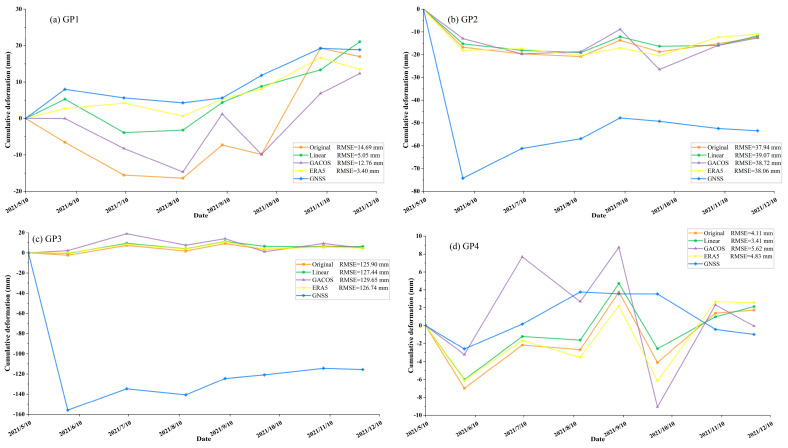

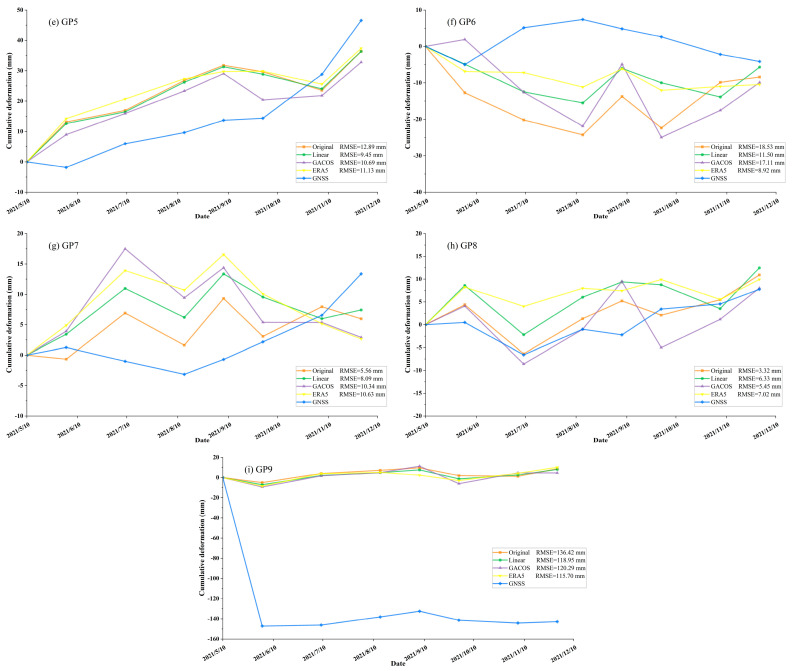
Comparison of the cumulative deformation before and after InSAR tropospheric delay correction and the cumulative deformation measured by the GNSS (**a**–**i**).

Figure 14 compares the deformation time series at the site without tropospheric delay correction, using the GNSS, and the three tropospheric delay correction methods (Linear, GACOS, and ERA5). The InSAR deformation values of stations such as Sites 2, 3, and 9 were considerably different from the GNSS monitoring deformation values. This was due to the landslide movement that occurred at the site location, resulting in the GNSS monitoring of deformation values greater than 100 mm, which is beyond the deformation monitoring range of InSAR (10–100 m/y) [40]. In addition, InSAR technology can obtain spatial continuous surface deformation monitoring results, which are the one-dimensional projections of real three-dimensional deformations in the LOS direction, while the GNSS can obtain three-dimensional deformation results with low spatial resolution. When monitoring a specific point where high gradient deformation occurs, the deformation results obtained by the GNSS are different from those obtained by the InSAR. Alternatively, we analyzed these sites by observing the trend of the deformation point–line diagram, which does not affect the results of the tropospheric delay estimation and accuracy verification. Table 2 shows the changes in the RMSE before and after the tropospheric delay correction. The average RMSE of the original cumulative deformation value is 39.93 mm, and the average RMSE after Linear, GACOS, and ERA5 correction was reduced to 36.59, 38.96, and 36.27 mm, respectively. This demonstrates that all three methods are effective in reducing the deformation error and that the correction effect of the ERA5 and Linear methods is greater. The methods improved the tropospheric delay at Sites 1, 5, 6, and 9. At the other sites, the residual error between the deformation value of each point monitored by the InSAR and the GNSS observation value increases after correction. This shows that there are spatial differences in InSAR deformation monitoring and inversion at the same time.

### 3.4. Study Area in Different Seasons

To further study the applicability of the three methods for tropospheric delay correction under different conditions, 32 interferograms with consistent time scales were selected from the 92 interferograms for analysis and processing. The information of the 32 interferograms is shown in Table 3.

**Figure 15:**
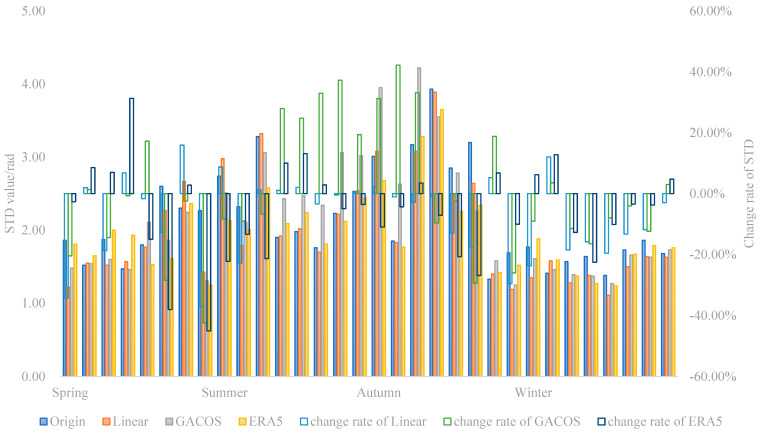
Correction effect diagram based on the three methods in different seasons. The solid bar represents the STD of each interferogram before and after correction, and the hollow bar represents its change rate.

Figure 15 and Table 4 show the correction results of the seasonal grouping for the sample, covering the entire study area. The STD of the interferogram before correction reasonably reflects the atmospheric activity in each season. From Figure 15, it can be seen that the atmospheric activity in the region is high in summer and autumn, and the STD value fluctuates at approximately 2.50 rad. In winter and spring, it was relatively stable, and the STD was lower than 2.00 rad. According to the statistics of Table 4, the average STD of the original phase of the 32 interferograms is 1.96 and 1.63, respectively, in the spring and winter when the atmospheric activity is stable, and the STD is reduced after the tropospheric delay correction by the three methods. Among them, the improvement rates of the three methods in spring are similar, with GACOS showing the best results, with an improvement rate of 15.30%. The Linear method has the highest improvement rate in winter, which is 12.27%. Meanwhile, in summer and fall, when the atmospheric convective activity is strong, the average STD of the original phase reaches 2.38 and 2.63. Among the three methods, GACOS shows poor correction results with improvement rates of −8.82% and 0.76%. ERA5 shows better correction results than the Linear method, with improvement rates of 10.92% and 12.93%, respectively. The average improvement rates of STD in spring, summer, autumn, and winter were 13.94%, 2.1%, 7.10%, and 7.57%, respectively.

**Figure 16:**
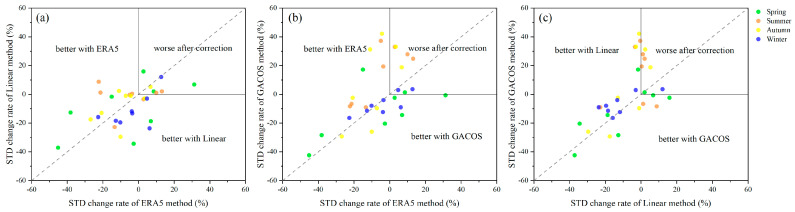
Comparison of the correction effects of the tropospheric delay correction methods. (**a**–**c**) represent the comparison of the standard deviation of the interference phase of the three correction methods. Different color points represent STDs in different seasons. Negative values indicate improvement, and positive values indicate the introduction of residuals or overcorrection.

Figure 16 compares the change in the rate of the standard deviation of the interference phase of the three correction methods by season. The upper right corner of each image shows the number of interferograms with invalid corrections. Among the 32 interferograms, the Linear, GACOS, and ERA5 methods showed correction effects in 21, 19, and 20 interferograms, respectively, and the standard deviation improvement rates were 14, 9, and 13 interferograms, which is greater than 10%. This means that less than half of the interferograms showed significant correction effects.

**Figure 17:**
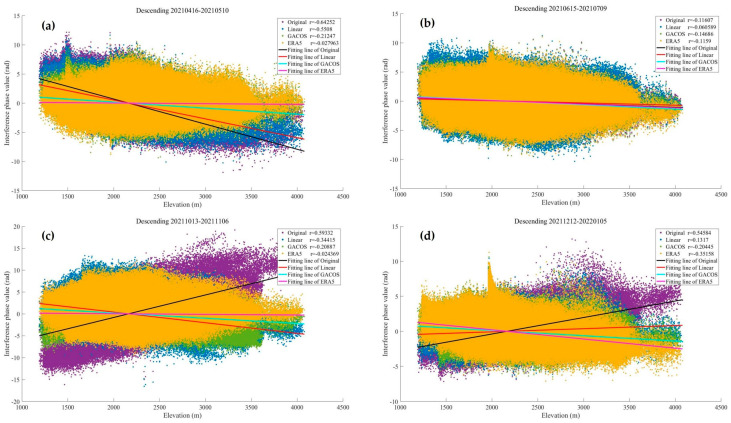
The relationship between the interferometric phase and elevation of the study area in the four seasons. The Pearson correlation coefficient between the two is calculated in the upper right corner of the figure: (**a**) 16 April to 10 May (in spring); (**b**) 15 June to 9 July (in summer); (**c**) 13 October to 6 November (in autumn); (**d**) 12 December to 5 January (in winter).

In order to further explore the changes in the atmospheric delay phase in the study area during the four seasons and the advantages and disadvantages of each method under different time conditions, we screened out the phase values in the interferograms that did not include obvious deformation signals based on Table 4. Subsequently, the relationship between the phase and elevation of the study area in the four seasons was visualized, and the Pearson correlation coefficient between the two was calculated. Figure 17 shows the statistical results of specific dates, which contain the obvious seasonal characteristics of the four seasons. It can be seen from this that the phase value of the original interferogram in summer does not change significantly with the elevation. The Pearson correlation coefficient is close to 0, and the atmospheric turbulence activity dominates. There is no significant change in the interferometric phase value after correction by the three methods, which does not diminish the influence of the tropospheric delay. In other seasons, the phase values show an obvious linear relationship with elevation, the Pearson correlation coefficient is greater than 0.5, and the vertical stratification part of the tropospheric delay in the interferogram is weakened by the corrections of the three methods.

**Figure 18:**
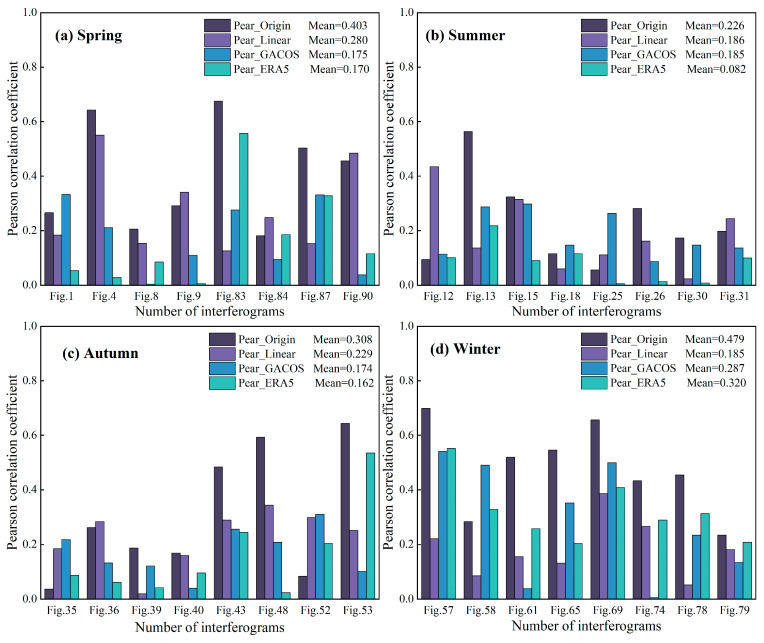
The change in the absolute value of the Pearson correlation coefficient between the interferometric phase and the elevation of the 32 interferograms before and after the tropospheric delay correction based on the three methods. (**a**–**d**) represent the statistics of spring, summer, autumn, and winter respectively, and the average value of the Pearson correlation coefficient in each season is calculated in the upper right corner of the figure.

Figure 18 shows the absolute value of the Pearson correlation coefficient between the phase and the elevation of each interferogram before and after tropospheric delay correction in the four seasonal periods. It can be seen more clearly that the correlation between the phase and the elevation in spring and winter is stronger than that in summer and autumn, and the time period with weak correlation is mostly concentrated in the late summer and early autumn. The Linear method has a stronger ability to weaken the vertical stratification delay in winter than the other two methods, and the ERA5 method shows a correction advantage, except in winter.

## 4. Discussion

The analysis of the four representative research areas in Section 3.2 shows that at the junction of the Hengduan Mountains and the Yunnan–Kweichow Plateau, the three methods are affected by different terrains and meteorological conditions, and their tropospheric delay correction effects are distinct. The Eryuan region in the north is significantly affected by the monsoon climate in the spring. However, the high altitude of the Hengduan Mountains in the central part of the region blocks the spread of warm air currents and forms a frontal system so the eastern basin of Eryuan, which is located in front of the fronts, is controlled by warm and humid air, while cold air is strengthened in the western alpine valley area, which is located behind the peaks. In spring, thunderstorms in the region often occur in the east, accompanied by turbulent signals, while in the west, the weather changes are more stable. At this time, the GACOS method, with its higher spatial resolution, can accurately distinguish the vertical stratification component in the west and the turbulence delay component in the east, and the tropospheric delay correction is relatively effective. In addition, in the spring when the meteorological conditions are more complex, the ERA5 method does not have this ability but still accurately identifies the vertical stratification component in the region with better stability. The Linear method, on the other hand, is not applicable under this condition due to its inability to estimate the turbulent mixing process caused by meteorological factors. The eastern Binchuan area was also affected by the monsoon in the spring, but due to the lack of continuous mountains in the region, no frontal system was generated, and warm and humid air flow affected the whole region. Under this condition, the ERA5 method, with its higher temporal resolution, is able to recognize the turbulent delayed signals for a short period of time with better correction. The Dali region in the south belongs to the alluvial plain terrain with low topography, which is significantly affected by the southwest monsoon in the summer, and the large lakes in the territory also complicate the water vapor resources of the region in the summer, with high rainfall and an uneven distribution. Here, the correction by each of the three methods has little effect and sometimes introduces additional errors. We speculate that the reason for this is that a variety of fast-varying, small-scale turbulent activities often occur in this region during this seasonal period, which require a higher spatial and temporal resolution for the tropospheric delay correction. Although the Linear method cannot correct the turbulent delay part in this case, the identification of the vertical stratification delay is still accurate, and it can correct the tropospheric delay at the hills and mountains in the Dali area. The Yangbi region in the west belongs to the Hengduan Mountain alpine valley area and has a large topographic relief, which is dry in winter with little rainfall, and the vertical stratification delay is dominant. However, since the region contains many ridge–valley terrain combinations and still has three-dimensional climate change characteristics, the correction effect of the Linear method is still not as good as that of the ERA5 method, and the GACOS method combined with the iterative model is not as stable as the ERA5 method combined with the atmospheric reanalysis data.

However, the factors that determine the correction effect are not singular. It can be seen from Section 3.4 that the region is affected by monsoons, and in the dry season (November–April), precipitation accounts for only 15% of the whole year. Dry conditions reduce the atmospheric convection activity; therefore, the correction effect of the tropospheric delay is best in winter and spring. In summer, there is a negative correction, which may be due to the subtropical plateau monsoon climate, which causes frequent local airflow disturbances in the study area. The water vapor content is more abundant, the water vapor change is more complex, and the tropospheric delay correction accuracy is low in this season compared with other seasons. In addition, the Yunnan–Kweichow Plateau is obviously affected by the Kunming quasi-stationary front, and its plateau-terrain-blocking effect causes the southward cold air to move slowly or even to stagnate [41]. Extreme weather phenomena such as rainstorms, hail, and thunderstorms occur in the period from the end of autumn to the beginning of spring. As described in Section 3.2.1, thunderstorms occur in the Binchuan area in the spring. We found that the tropospheric delay is closely related to rainstorm timing. During the period before the rainstorm, the atmospheric precipitable water vapor (PWV) begins to accumulate, resulting in a sudden increase in the zenith tropospheric delay (ZTD), indicating the arrival of the rainstorm. After the rainstorm, the PWV decreases, and the ZTD tends to be stable [42]. This causes the tropospheric delay correction effect, particularly in the GACOS model correction, to fluctuate throughout the season.

Less than half of the interferograms showed significant correction effects. The main reason for this is that, owing to the limitations of the spatial and temporal resolution and model accuracy, the three methods are not sufficient to correct the tropospheric delay caused by the turbulence effect at a local scale. ERA5 and GACOS products with ECMWF atmospheric physical parameters are not suitable for estimating turbulence models using a single interferogram [43]. The imaging time of the descending SAR data used in this study was 23:21 coordinated universal time (UTC), which converted to 7:21 a.m. China standard time (CST). At this time, the molecular activity frequency in the atmosphere was low, and the atmosphere was relatively stable. Therefore, some interferograms have been overcorrected [44]. From the results of the comparison of the three methods, the Linear and the ERA5 methods have similar tropospheric delay correction effects in this area. The Linear method uses a simple linear relationship to fit the tropospheric delay associated with topography, which cannot reflect the turbulent atmospheric characteristics and effects. The ERA5 method fuses ground observations, balloons, satellites and other observations of the atmospheric state through data assimilation techniques, and after optimization and adjustment of the initial field and boundary conditions, the model forecasts are closer to the real atmosphere. The former has an absolute advantage over the other two methods in winter. The reason is that the meteorological conditions in this region are stable in winter, resulting in less turbulent activity in the atmosphere and a strong correlation between the tropospheric delay phase and the elevation. Moreover, the study area is large. The above two factors lead to more accurate correlation coefficient estimates of K from the empirical linear model in the Linear method. The latter, by combining atmospheric reanalysis datasets with high temporal and spatial resolution, not only identifies local turbulence variations during the summer and autumn time periods, but also accurately estimates terrain-related delays through the correlation of meteorological parameters with the elevation. In spring, due to the influence of abnormal anticyclonic circulation, the entire troposphere sinks and is prone to drought, and the total amount of the atmospheric water vapor is small. However, weather conditions such as strong convection or frontal systems can still occur in some regions, leading to regional climate characteristics. GACOS combines the atmospheric parameters of ECMWF products with high spatial resolution and uses the iterative tropospheric decomposition model to separate the vertical stratification delay and the turbulent mixing delay from the total tropospheric delay, which can capture the delay signals of medium–long wave and medium–short wave [45]. Therefore, in spring, the GACOS method is superior to the other two methods because it can identify the turbulence change in the atmosphere in a small area.

## 5. Conclusions

In this study, based on the spatial and temporal differences of tropospheric effects, the boundary area of the Hengduan Mountains and Yunnan–Kweichow Plateau, which is significantly affected by the subtropical monsoon climate, was taken as the research area. Different seasons and landforms covered in 25 Sentinel-1A images were selected as the experimental dataset. The applicability of three tropospheric delay correction methods, Linear, GACOS, and ERA5, at different times and in different spaces was evaluated and analyzed by using phase standard deviation, elevation phase correlation, and GNSS monitoring results.

Based on the above evaluation results, we conclude that:

1. The Linear method has a good effect in winter with stable convective activity and is suitable for large-scale alpine and gorge areas. However, it is limited to the estimation of vertical stratification delay, which is not applicable in areas with an active atmosphere or a flat terrain.
2. GACOS is not suitable as an overall correction method for estimating and correcting the tropospheric delay in a large area. It is more suitable for correcting the tropospheric delay for time periods or small regions where there are regional climate changes.
3. The ERA5 method provides good correction results in spring, summer, and autumn in the subtropical monsoon climate zone. The high temporal and spatial resolution of the ERA5 dataset ensures the accuracy of the tropospheric delay correction under different temporal and spatial conditions, provided there are no fast-changing turbulent motion trends in the atmosphere. In addition, the ERA5 method is more effective in correcting areas with a moderate terrain relief than in areas with flat or complex terrain.

In summary, each method has its inherent advantages and disadvantages, and they have different sensitivities to different components of atmospheric delay. To improve the monitoring accuracy of time-series InSAR in the low-latitude plateau canyon area, which is significantly influenced by the subtropical monsoon climate, it is necessary to select a suitable tropospheric delay correction method by comprehensively taking into account the characteristics of the temporal and spatial variability. For time periods and regions with different seasons and terrain, the above findings need to be taken into account to refine the use of tropospheric delay methods. At the same time, the fast-changing and unstable turbulent motion in the atmosphere of this region requires higher temporal and spatial resolution products for observation, and the tropospheric delay correction methods still need to be improved. The current large number of InSAR tropospheric delay correction methods lack a means of reliable quality control, and it is a future research direction to develop some criteria for defining the uncertainty in atmospheric delays, as well as to address the problem of error propagation in the InSAR time-series deformation results. This requires a comprehensive variance–covariance matrix to assess the impact of the various sources of error in the InSAR. In addition, the current research in the field of tropospheric delay correction is mainly further discussed and analyzed by defining non-deformed regions. The research on tropospheric delay correction in areas with long time baselines or obvious deformation signals is not perfect. How to effectively separate deformation signals, atmospheric delay signals, and other noise to improve the monitoring accuracy of the InSAR is still the main problem to be solved in the future.

## Figures and Tables

**Table 1:** Sentinel—1A data parameters.

Tracks	Number of Images	Time Coverage	Imaging Time (UTC)	Number of Interferograms
Descending	25	4 April 2021–23 April 2022	23:14	92

**Table 2:** RMSE changes before and after tropospheric delay correction.

GNSS Point	RMSE of	RMSE of	RMSE of	RMSE of
	Original	Linear	GACOS	ERA5
1	14.69	5.05	12.76	3.40
2	37.94	39.07	38.72	38.06
3	125.90	127.44	129.65	126.74
4	4.11	3.41	5.62	4.83
5	12.89	9.45	10.69	11.13
6	18.53	11.50	17.11	8.92
7	5.56	8.09	10.34	10.63
8	3.32	6.33	5.45	7.02
9	136.42	118.95	120.29	115.70
Mean	39.93	36.59	38.96	36.27

**Table 3:** Details of the selected interferograms.

Number of Interferograms	Season	Time Coverage	Temporal Baseline (day)
FIG. 1, 4, 8, 9, 83, 84, 87, 90	Spring	22 February–3 June	101
FIG. 12, 13, 15, 18, 25, 26, 30, 31	Summer	3 June–26 August	84
FIG. 35, 36, 39, 40, 43, 48, 52, 53	Autumn	26 August–30 November	96
FIG. 57, 58, 61, 65, 69, 74, 78, 79	Winter	30 November–22 February	84

FIG. in the table indicates the selected interferogram number.

**Table 4:** The average STD and improvement rate of interferograms in the four seasons before and after correction by the three methods.

Season	Evaluation of Correction	Original	Linear	GACOS	ERA5
Spring	Average of Phase Standard Deviation of All Figures	1.96	1.73	1.66	1.67
	Improvement Rate	-	11.73%	15.30%	14.80%
Summer	Average of Phase Standard Deviation of All Figures	2.38	2.28	2.59	2.12
	Improvement Rate	-	4.20%	−8.82%	10.92%
Autumn	Average of Phase Standard Deviation of All Figures	2.63	2.43	2.61	2.29
	Improvement Rate	-	7.60%	0.76%	12.93%
Winter	Average of Phase Standard Deviation of All Figures	1.63	1.43	1.52	1.57
	Improvement Rate	-	12.27%	6.75%	3.68%

## Data Availability

Data are contained within the article.
